# Supplementary material for: Linking longitudinal and cross-sectional biomarker data to understand host-pathogen dynamics: Leptospira in California sea lions (Zalophus californianus) as a case study
Source: PLoS Negl Trop Dis. 2020 Jun 29;14(6):e0008407. doi: 10.1371/journal.pntd.0008407 (PMC7351238; doi:10.1371/journal.pntd.0008407)
Supplement: S1 Table — In parentheses are the number of animals shedding leptospires for each log2 antibody titer level over the total number of PCR tested animals. (DOCX) [file pntd.0008407.s001.docx]

**S1 Table.** Total number of sea lions with a given log_2_ antibody titer by year for wild-caught (WILD), stranded (STRAND) and subclinically infected (SUB1 and SUB2) sea lions. In parentheses are the number of animals shedding leptospires for each log_2_ antibody titer level over the total number of PCR tested animals.

|  | WILD | | | | | | | STRAND | | | | | | | SUB1 | | | SUB2 | | |
| --- | --- | --- | --- | --- | --- | --- | --- | --- | --- | --- | --- | --- | --- | --- | --- | --- | --- | --- | --- | --- |
| Titer | | 10 | 11 | 12 | 13 | 14 | Total | | 10 | 11 | 12 | 13 | 14 | Total | | 11 | 12 | | 11 | 12 |
| 0 | | 79 (1/41) | 79 (5/70) | 108 (21/93) | 142 (0/134) | 260 (0/251) | 668 (27/589) | | 100 (0/13) | 38 (2/12) | 40 (1/10) | 49 (0/18) | 114 (0/22) | 341 (3/75) | | - | 1 | | - | - |
| 1 | | - | - | - | - | 6 (0/6) | 6 (0/6) | | - | 1 | 2 (0/1) | - | 3 | 6 (0/1) | | - | 5 | | - | - |
| 2 | | - | - | - | - | 5 (0/5) | 5 (0/5) | | 3 (0/1) | - | 3 | 3 (0/1) | 1 | 10 (0/2) | | - | 1 | | - | - |
| 3 | | - | 1 (1/1) | 1 (1/1) | 1 (0/1) | 3 (0/3) | 5 (2/6) | | 1 | 1 (0/1) | 2 (0/2) | 3 (1/3) | 1 (0/1) | 8 (1/7) | | - | 2 | | - | 1 |
| 4 | | - | 1 (1/1) | 1 (0/1) | - | 2 (0/2) | 4 (1/4) | | - | 2 (0/1) | - | 3 (0/3) | - | 5 (0/4) | | 1 (1) | - | | - | 4 |
| 5 | | - | 1 (1/1) | - | - | - | 1 (1/1) | | - | 1 (0/1) | - | 2 (0/2) | 2 (0/1) | 5 (0/4) | | - | - | | 1 | 2 |
| 6 | | 2 (2/2) | 1 (1/1) | 1 (1/1) | - | - | 4 (4/4) | | - | 2 (0/1) | - | 1 (0/1) | - | 3 (0/2) | | - | - | | - | - |
| 7 | | 1 (1/1) | 4 (3/4) | - | - | - | 5 (4/5) | | - | 2 (1/1) | - | - | - | 2 (1/1) | | 2 | - | | 1 | - |
| 8 | | - | 1 (1/1) | 1 (1/1) | - | - | 2 (2/2) | | 2 | 3 (1/1) | 1 | 1 | - | 7 (1/1) | | - | - | | - | - |
| 9 | | - | 3 (2/3) | - | - | - | 3 (2/3) | | 3 | 9 (2/3) | 5 (1/1) | - | - | 20 (4/7) | | - | - | | 1 | - |
| 10 | | 1 (1/1) | 3 (3/3) | 1 (1/1) | - | - | 5 (5/5) | | 8 (3/4) | 23 (7/8 | 7 | - | - | 38 (10/12) | | - | - | | 1 | - |
| 11 | | 1 | 2 (2/2) | - | - | - | 3 (2/2) | | 3 (1/1) | 60 (14/14) | 4 (0/1) | - | - | 67 (12/13) | | - | - | | - | - |
| 12 | | - | 3 (3/3) | 1 (1/1) | - | - | 4 (4/4) | | 5 (2/2) | 41 (9/9) | 1 (1/1) | - | - | 47 (12/12) | | - | - | | - | - |
| 13 | | 1 (1/1) | - | - | - | - | 1 (1/1) | | 3 (1/1) | 10 (4/4) | 2 | - | - | 15 (5/5) | | - | - | | - | - |
| 14 | | - | - | - | - | - | - | | 1 | 2 (1/1) | 1 (1/1) | - | - | 4 (2/2) | | - | - | | - | - |
