# Supplementary material for: Linking longitudinal and cross-sectional biomarker data to understand host-pathogen dynamics: Leptospira in California sea lions (Zalophus californianus) as a case study
Source: PLoS Negl Trop Dis. 2020 Jun 29;14(6):e0008407. doi: 10.1371/journal.pntd.0008407 (PMC7351238; doi:10.1371/journal.pntd.0008407)
Supplement: S2 Table — (DOCX) [file pntd.0008407.s002.docx]

**Table S2**. Predicted shedding duration by titer level assuming a constant titer decay rate of 0.058 log2 antibody titer units/day (the median decay rate of the CLINICAL animals) and an initial titer of 11 (the median initial titer of the CLINICAL animals).

| Titer | Days |
| --- | --- |
| 0 | 189 |
| 1 | 171 |
| 2 | 154 |
| 3 | 137 |
| 4 | 120 |
| 5 | 103 |
| 6 | 86 |
| 7 | 69 |
| 8 | 51 |
| 9 | 34 |
| 10 | 17 |
| 11 | 0 |
| 12 | 0 |
| 13 | 0 |
| 14 | 0 |
